# Cytokine signaling convergence regulates the microglial state transition in Alzheimer’s disease

**DOI:** 10.1007/s00018-021-03810-0

**Published:** 2021-04-13

**Authors:** Shun-Fat Lau, Amy K. Y. Fu, Nancy Y. Ip

**Affiliations:** 1 Division of Life Science, State Key Laboratory of Molecular Neuroscience, Molecular Neuroscience Center, The Hong Kong University of Science and Technology, Clear Water Bay, Hong Kong, China; 2 Hong Kong Center for Neurodegenerative Diseases, Hong Kong, China; 3 Guangdong Provincial Key Laboratory of Brain Science, Disease and Drug Development, HKUST Shenzhen Research Institute, Shenzhen–Hong Kong Institute of Brain Science, Shenzhen, 518057 Guangdong China

**Keywords:** Interleukin, Chemotaxis, Phagocytosis, Amyloid, Tau

## Abstract

Genetic analyses have revealed the pivotal contribution of microglial dysfunctions to the pathogenesis of Alzheimer’s disease (AD). Along AD progression, the accumulation of danger-associated molecular patterns (DAMPs) including beta-amyloid and hyperphosphorylated tau continuously stimulates microglia, which results in their chronic activation. Chronically activated microglia secrete excessive pro-inflammatory cytokines, which further regulate microglial responses towards DAMPs. This has spurred longstanding interest in targeting cytokine-induced microglial responses for AD therapeutic development. However, the cytokine-induced microglial state transition is not comprehensively understood. Cytokines are assumed to induce microglial state transition from a resting state to an activated state. However, recent evidence indicate that this microglial state transition involves multiple sequential functional states. Moreover, the mechanisms by which different functional states within the cytokine-induced microglial state transition regulate AD pathology remain unclear. In this review, we summarize how different cytokine signaling pathways, including those of IL-33 (interleukin-33), NLRP3 inflammasome–IL-1β, IL-10, and IL-12/IL-23, regulate microglial functions in AD. Furthermore, we discuss how the modulation of these cytokine signaling pathways can result in beneficial outcomes in AD. Finally, we describe a stepwise functional state transition of microglia induced by cytokine signaling that can provide insights into the molecular basis of the beneficial effects of cytokine modulation in AD and potentially aid therapeutic development.

## Introduction

Alzheimer’s disease (AD), the most prevalent form of dementia, is characterized by beta-amyloid (Aβ) deposition and neurofibrillary tangle accumulation [1–3]. The accumulation of these danger-associated molecular patterns (DAMPs) triggers microglial activation in which activated microglia first migrate towards DAMPs and mediate their clearance through phagocytosis [4, 5]. Activated microglia simultaneously secrete cytokines and immune signaling molecules such as interleukins (ILs) and reactive oxygen species [6, 7]. However, as AD progresses, microglia fail to clear the excess accumulation of DAMPs, resulting in chronic activation and excessive cytokine secretion in the brain.

Therefore, targeting cytokines milieus in the brain has long been a goal of AD research. However, it remains unclear how cytokine signaling should be modulated in AD to yield beneficial outcomes. One major obstacle is that cytokine-induced microglial responses can elicit both beneficial and detrimental effects. Therefore, it is essential to comprehensively understand cytokine-induced microglial activation in AD.

In this review, we summarize the functional roles of cytokine signaling in mediating the responses of microglia and their communication with other cell types. We also review some of the cytokine signaling pathways that have potential therapeutic value for AD, including those of IL-33, NLRP3 inflammasome–IL-1β, IL-12/IL-23, and IL-10. Finally, we discuss and propose how cytokines stimulate a stepwise state transition of microglia that leads to the amelioration of AD pathology.

## Neuroinflammation and microglial activation in Alzheimer’s disease

Neuroinflammation is characterized by elevated cytokine levels in the central nervous system microenvironment. In AD, the levels of certain cytokines such as IL-1β and TNF-α (tumor-necrosis factor alpha) increase after the accumulation of Aβ but before the onset of cognitive impairment, suggesting that neuroinflammation contributes to AD pathogenesis early on [8–11]. As microglia are the primary immune effectors, their functions are greatly influenced by elevated cytokine levels in AD [12, 13]. Accordingly, elevated cytokine levels can regulate various microglial responses and potentially regulate AD pathogenesis.

Cytokine signaling is essential for the regulation of microglial responses to Aβ pathology. Initial Aβ accumulation triggers microglial activation and cytokine secretion, which in turn stimulate other microglia to react to Aβ. As such, several cytokines including IL-1β and IL-33 promote microglial activation and mediate Aβ clearance [14, 15]. This suggests that microglial activation, which is induced by transient increases in cytokine levels, can regulate Aβ clearance and hence incur beneficial effects.

However, microglial activation also incurs adverse effects due to the chronic elevation of cytokine levels. Chronic microglial activation can result in the phagocytosis of non-Aβ materials including synapses and astrocytic processes, which lead to synaptic loss and blood–brain barrier breakdown, respectively [16–18]. Moreover, a recent study demonstrates that increased levels of microglia-derived cytokines including IL-1α and TNF-α drive the state transition of astrocytes towards a neurotoxic form that mediates neuronal death [19]. Therefore, the duration of cytokine-induced microglial response (i.e., transient vs. chronic) is the distinguishing feature of the functional roles of microglia in AD pathogenesis.

## Cytokine signaling pathways as therapeutic targets for Alzheimer’s disease

Given the role of chronic neuroinflammation in AD pathogenesis, targeting cytokine milieus to modulate microglial functions is an attractive therapeutic approach for AD. Indeed, there have been clinical trials targeting general cytokine milieus using sargramostim [20], a small molecule RAGE inhibitor [21], NSAIDs [22, 23], and etanercept [24] in patients with AD or mild cognitive impairment [25]. However, most of them were aborted owing to a lack of efficacy, suggesting that simply inhibiting the secretion of pro-inflammatory cytokines cannot effectively restore brain homeostasis or improve cognitive performance in patients with AD.

These failures also underscore our incomplete understanding of the roles and immunomodulatory functions of specific cytokine signaling pathways in AD. This knowledge gap is partly due to the lack of unbiased, comprehensive profiling of different cytokines along with AD progression. While previous studies using candidate approaches show that dysregulated levels of several specific cytokines including IL-33, NLRP3 inflammasome, IL-12/IL-23, and IL-10 are associated with AD pathogenesis [15, 26–30], modulating these cytokine signaling pathways in mouse models of amyloidosis and hyperphosphorylated tau results in beneficial outcomes. Accordingly, below we review the beneficial effects of modulating these cytokine signaling pathways in AD.

### IL-33 replenishment ameliorates Alzheimer’s disease pathology by promoting a PU.1–dependent microglial state transition

IL-33 is an IL-1 family member that functions as an alarmin to trigger an immune response to cellular damage or injury [31, 32]. Interestingly, IL-33 is most abundant in the brain and lungs [33]. In the brain, IL-33 is enriched within the nuclei of oligodendrocytes but is also found in astrocytes and neurons [33, 34]. Nuclear IL-33 is inactive and interacts with histones [35]. Upon central nervous system injury, IL-33 is rapidly released by necrotic or apoptotic cells and simulates microglial recruitment [31, 33, 36, 37].

Genetic studies demonstrate that AD pathogenesis is associated with single nucleotide polymorphisms of IL-33 and its receptor ST2 [38, 39]. Specifically, patients with AD and mild cognitive impairment exhibit reduced gene expression of *IL33* and increased serum levels of soluble ST2 (the decoy receptor of IL-33), respectively [15, 39]. This suggests that IL-33/ST2 signaling is impaired in AD. Concordantly, IL-33 deficiency can drive tau pathology and neuronal loss in aged mice [40]. In addition, in the APP/PS1 mouse model of amyloid deposition, IL-33 administration can ameliorate synaptic impairment, improve cognitive performance, and reduce levels of Aβ and pro-inflammatory cytokines (e.g., IL-1β, IL-6, and NLRP3 inflammasome) [15]. These findings collectively show that restoring IL-33/ST2 signaling can alleviate AD pathology.

Microglia mediate these beneficial outcomes of replenishing IL-33 in AD. IL-33 administration triggers the state transition of microglia and induces a subpopulation of IL-33–responsive microglia that have a distinct transcriptomic signature characterized by increased expression of major histocompatibility complex class II genes and homeostatic signature genes as well as enhanced Aβ-phagocytic and clearance capacity [26]. Furthermore, epigenetic analyses show that IL-33 can remodel the chromatin accessibility and PU.1-binding landscapes in microglia, resulting in the induction of IL-33–responsive microglia. These lines of evidence delineate the molecular mechanisms of the IL-33–induced microglial state transition and its beneficial outcomes. However, given that PU.1 functions as a lineage-dependent transcription factor and is unlikely to be direct downstream of IL-33/ST2 signaling, further investigation is required to understand how PU.1 cooperates with transcription factors downstream of IL-33 to mediate the induction of IL-33–responsive microglia. Nonetheless, these findings show that restoring IL-33/ST2 signaling in AD promotes the clearance activity of microglia via PU.1-dependent transcriptional control, which yields beneficial outcomes.

### NLRP3 inflammasome activation exacerbates Aβ and tau pathology

NLRP3 inflammasome is a multiprotein complex that consists of the sensor NLRP3 (NACHT-, LRR-, and pyrin [PYD] domain-containing protein 3), adaptor ASC, and effector pro-caspase-1 [41]. NLRP3 inflammasome acts a cytosolic pattern-recognition receptor that mediates the sensing of DAMPs such as Aβ [41, 42]. Upon activation by Aβ, NLRP3, ASC, and pro-caspase-1 self-assemble, which results in the cleavage of pro-caspase-1 into proteolytically active caspase-1 [43]. In turn, this active caspase-1 can cleave pro-IL-1β and pro-IL-18 to generate the mature forms of IL-1β and IL-18, respectively. Meanwhile, inhibiting caspase-1 activity attenuates Aβ-induced IL-1β production in microglia [43]. These findings show that NLRP3 plays an important role in the regulation of IL-1β production by microglia in AD.

Besides controlling the release of cytokines, NLRP3 inflammasome contributes to other aspects of AD pathogenesis. First, NLRP3 contributes to amyloid pathology by regulating the phagocytic capacity of microglia in AD. Genetic ablation of NLRP3 in APP/PS1 mice enhances the Aβ-phagocytic capacity of microglia, which reduces the level of Aβ in the brain [27]. This coincides with the induction of anti-inflammatory microglial phenotypes characterized by increased gene expression of *Arg1*, *Fizz1*, *Il4*, and *Nos2.* However, it remains unclear how these anti-inflammatory microglia contribute to the enhanced phagocytic capacity of microglia. Second, activated microglia can secrete ASC, a component of NLRP3 inflammasome, to bind and cross-seed Aβ peptides to form Aβ plaques [44]. Once released into the extracellular space, ASC can bind Aβ peptides to form the core of an Aβ deposit and facilitate further aggregation of Aβ. This effect of ASC-mediated Aβ seeding also contributes to the accumulation of Aβ deposition in AD. Finally, NLRP3 inflammasome activation also regulates tau pathology in AD [45]. The reduction of NLRP3 activation in a mouse model of tau pathology is associated with reduced activity of CaMKIIα and GSK3β (kinases that phosphorylate tau) and increased activity of PP2A (a phosphatase that dephosphorylates tau) [46]. Thus, reducing NLRP3 inflammasome activation contributes to the overall reduction in tau pathology and improves cognitive performance in mice.

These findings collectively demonstrate that the action of NLRP3 inflammasome in AD contributes to amyloid and tau pathology through the modulation of the clearance activities of microglia, Aβ seeding, and kinase activities.

### IL-12/IL-23 inhibition alleviates Aβ pathology in a sex-specific manner

IL-12 and IL-23 structurally share the same IL-12p40 protein subunit, which links to the IL-12p35 and IL-23p19 subunits, respectively [47, 48]. While IL-12/IL-23 dysregulation has long been considered to drive the pathogeneses of several autoimmune diseases such as experimental autoimmune encephalomyelitis and arthritis [47, 49], recent evidence shows that dysregulated IL-12/IL-23 signaling can also contribute to AD. In patients with AD, IL-12p40 is elevated in the cerebrospinal fluid [28], which is associated with impaired cognitive performance. Thus, aberrant IL-12/IL-23 signaling contributes to AD pathogenesis, suggesting that its inhibition might produce beneficial outcomes in AD. Indeed, inhibiting IL-12/IL-23 signaling either by the administration of an IL-12p40–neutralizing antibody or genetic ablation of IL-12/IL-23 subunits ameliorates Aβ pathology and cognitive impairment in APP/PS1 mice [28]. Interestingly, a recent study also shows that the beneficial effect of inhibiting IL-12/IL-23 exhibits a sex-specific bias in APP23 transgenic mice [50]. While genetic ablation of IL-12p40 reduces the total Aβ level in male APP23 mice, it only reduces the soluble Aβ_1–40_ level in female APP23 mice. However, it remains unclear how sex contributes to this sex-specific beneficial effect of IL-12/IL-23 inhibition in AD. More importantly, the IL-12/IL-23 receptor gene *Il12rb1* is predominately expressed in oligodendrocytes and other non-myeloid cells [28, 51]. Therefore, it is unclear how inhibiting IL-12 and/or IL-23 signaling elicits beneficial effects through the regulation of the cell-state transition of non-myeloid cells in AD. Nonetheless, these findings collectively demonstrate that inhibiting IL-12/IL-23 signaling in AD can alleviate amyloid pathology in a sex-specific manner.

### IL-10 inhibition alleviates Aβ pathology through the regulation of microglial activation status

IL-10 is known for its role in the suppression of immune responses in peripheral immunity and is, therefore, considered an anti-inflammatory cytokine. IL-10 suppresses the secretion of pro-inflammatory cytokines including TNF-α, inflammasome activation, and the induction of major histocompatibility complex class II and co-stimulatory molecules in macrophages and dendritic cells [52–54]. The immunosuppressive effects of IL-10 are partly mediated through the activation of STAT3-dependent transcriptional control by its receptor, IL-10 receptor [55]. Thus, IL-10 plays a key role in restraining aberrant immune activation and dysregulated signaling associated with various immune-related diseases including AD.

Genetic analyses of patients with AD have identified that IL-10 promoter polymorphism is associated with AD risk in Chinese and Italian cohorts, suggesting that dysregulated IL-10 signaling potentially contributes to AD [56, 57]. Building on this finding, one study demonstrates that genetic ablation of IL-10 in APP/PS1 mice ameliorates Aβ pathology by promoting microglial activation [29]. Furthermore, subsequent transcriptome analysis showed that IL-10 ablation suppresses the gene signature of disease-associated microglia (i.e., *Apoe*, *Clec7a*, *Itgax*, and *Trem2*), probably due to the reduced Aβ level in the brain. On the other hand, IL-10 overexpression mediated by adeno-associated virus exacerbates Aβ pathology and cognitive impairment in TgCRND8 mice, another mouse model that overexpresses mutant human APP [30]. IL-10 overexpression stimulates ApoE (apolipoprotein E) production and aggregation with Aβ, thus inhibiting microglial Aβ phagocytosis. These findings collectively demonstrate that inhibiting IL-10 signaling ameliorates AD pathology through the activation of microglia.

### Cytokine signaling convergence and competition in Alzheimer’s disease

One intriguing observation stemming from the abovementioned findings is that all modulation of cytokine signaling—activation of IL-33, or inhibition of NLRP3 inflammasome–IL-1 or IL-10—promotes microglial activation and ameliorates Aβ pathology in AD (Table 1). While activating IL-33 signaling promotes microglial activation, why does inhibiting cytokine signaling elicit a similar effect? To answer this, we must first understand the interplay among cytokine signaling pathways within microglia. Here, we use IL-33, NLRP3 inflammasome–IL-1β, and IL-10 signaling to illustrate such signal interplay in microglia; we excluded IL-12/IL-23 signaling because microglia are not the primary target cells that respond to the modulation of these two cytokines.

**Table 1:** Summary of the beneficial effects of modulating specific cytokine signaling pathways in Alzheimer’s disease

Cytokine signaling	Receptor	Dysregulation in Alzheimer’s disease	Beneficial modulation	Functional changes in microglia	Molecular changes in microglia	Beneficial outcomes
IL-33	ST2 and IL-1RAP	↑ Soluble ST2 in serum [15] ↓ IL-33 expression in the brain [39]	IL-33 injection	↑ Aβ chemotaxis [26] ↑ Aβ phagocytosis [15, 26]	Induction of a subpopulation of MHC-II^+^ IL-33–responsive microglia [26] Epigenetic landscape reprogramming (i.e., chromatin accessibility and PU.1 binding) [26]	↓ Aβ level [15] ↓ Pro-inflammatory cytokine level [15] ↑ Synaptic plasticity and cognitive performance [15]
NLRP3 inflammasome–IL-1β	IL-1R and IL-1RAP	↑ NLRP3 inflammasome activation [27]	Genetic ablation of NLRP3 and ASC	↑ Aβ phagocytosis [27]	N/A	↓ Aβ level [27] ↓ Tau phosphorylation [45] ↓ Pro-inflammatory cytokine level [27] ↑ Synaptic plasticity and cognitive performance [27]
IL-10	IL-10R	N/A	Genetic ablation of IL-10 [29]	↑ Activation [29]	↓ Disease-associated microglial signature genes [29]	↓ Aβ level [29] ↑ Synaptic plasticity and cognitive performance [29]
IL-12/IL-23	IL-12R and IL-23R	↑ Cerebrospinal fluid IL-12p40 [50]	Genetic ablation of IL-12p40 and IL-12R [50]	N/A	N/A	↓ Total Aβ level in males [50] ↓ Aβ_1–40_ level in females [50]

*Aβ* Beta-amyloid

**Fig. 1:**
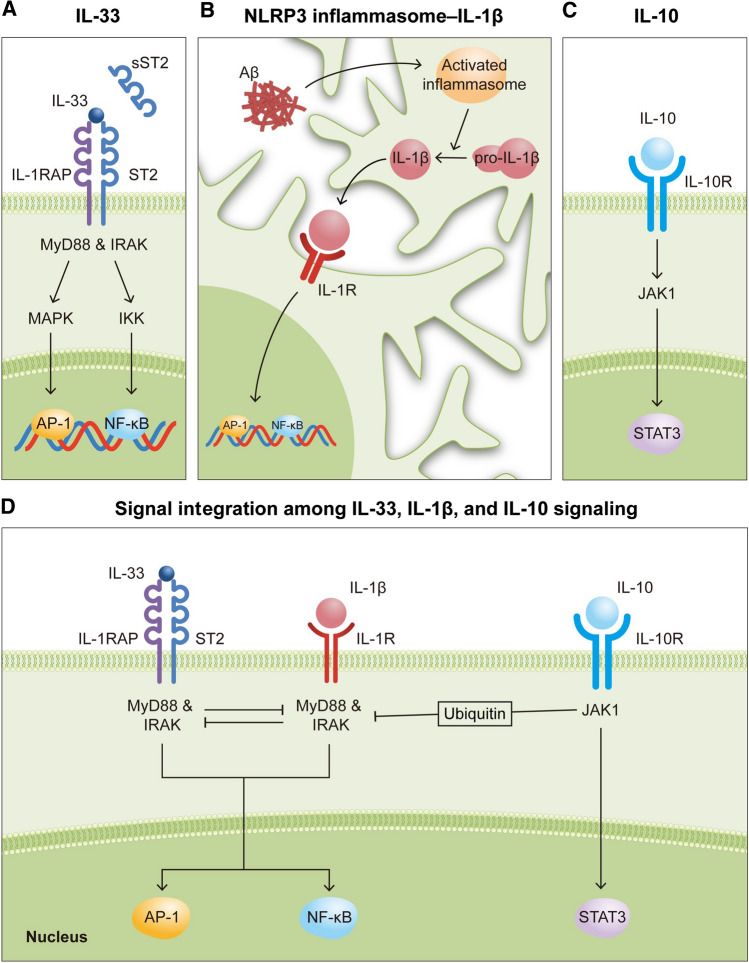
Interplay among cytokine signaling pathways within microglia. **a**–**c** Diagrams illustrating the signaling pathways downstream of IL-33 (**a**), NLRP3 inflammasome–IL-1β (**b**), and IL-10 (**c**). **d** Diagram illustrating the inhibitory effects of the simultaneous activation of multiple cytokine signaling pathways on neighboring pathways. Aβ, beta-amyloid

All three cytokine signaling pathways—IL-33, NLRP3 inflammasome–IL-1β, and IL-10—converge during signal transduction steps. For example, IL-33 triggers its downstream signaling by binding to ST2 and IL-1RAP heterodimers (Fig. 1a). This dimerizes the cytosolic adaptor protein MyD88 along with IRAK (IL-1R–associated kinase) to activate transcriptional control dependent on both AP-1 (activator protein 1) and NF-κB, which closely resembles the classical IL-1 signaling cascade [32]. Similar to IL-33, IL-1 signaling is one of the downstream signaling pathways of NLRP3 inflammasome activation. This is because activated NLRP3 inflammasome produces mature IL-1β for secretion. Once secreted, IL-1β acts on microglia via IL-1R to trigger the IL-1 signaling cascade (Fig. 1b) [58]. While IL-10 activates distinct sets of downstream signaling pathways (i.e., STAT3 via JAK1) [59], IL-10 activation can induce the ubiquitination of IRAK and TRAF, which are core signal transducers of the IL-1 signaling cascade, to limit IL-1–dependent responses (Fig. 1c) [60]. Hence, these findings collectively illustrate how the cytokine signaling pathways interact and converge at the signal transduction stage within microglia.

Because of this signaling convergence, the simultaneous activation of various cytokine signaling pathways due to the chronically elevated cytokine levels in AD triggers competition for the same set of intracellular signal transducers, which leads to mutual inhibition (Fig. 1d). This phenomenon is prominent in the peripheral immune response. For example, given that IL-33 and IL-1 employ the same downstream signaling cascade, the activation of ST2 signaling can inhibit IL-1R signaling or vice versa. Indeed, compared to control mice, myeloid cells from ST2-deficient mice produce significantly more pro-inflammatory cytokines when systemically challenged with IL-1 [61]. Besides myeloid cells, natural killer cells from ST2-deficient mice also exhibit enhanced production of pro-inflammatory cytokines such as IFN-γ [62]. Therefore, we hypothesize that suppressing pro-inflammatory cytokine signaling (e.g., that of NLRP3 inflammasome–IL-1β) relieves its inhibition of the signaling pathways of other cytokines (e.g., IL-33) and hence promotes beneficial microglial activation in AD (Fig. 1d).

Besides the abovementioned signaling competition, there are other possible negative feedback mechanisms within the cytokine signaling cascade. At the signal transduction level, IRAK-M, an inactive kinase downstream of Toll-like receptor, can inhibit the receptor signaling in macrophages by dissociating the IRAK signaling complex [63]. Also, SIGIRR, an IL-1R–related surface receptor, can inhibit IL-1 signaling by sequestering downstream signaling molecules [64]. Furthermore, at the transcriptional level, exaggerated cytokine response can be limited by stimulating the gene expression of decoy receptors and receptor antagonists upon cytokine activation. For example, IL-33 stimulates the expression of soluble ST2 (an IL-33 decoy receptor), and IL-1RA (an IL-1 receptor antagonist) to limit further cytokine activation [65, 66]. Therefore, when developing therapeutic strategies for AD based on the modulation of cytokine signaling, whether feedback mechanisms in cytokine signaling affect the expected beneficial outcome (e.g., microglial activation) must be considered. Accordingly, we propose targeting the cytokine inhibitory signal—for example by cytokine competition or decoy receptors of cytokines—as an alternative approach to promote the beneficial functions of microglia in AD.

## Stepwise microglial state transition upon cytokine activation in Alzheimer’s disease

The association between microglial activation and AD has been reported in patients for decades. However, its molecular basis was only recently elucidated through the advancement of single-cell transcriptome analysis. Studies profiling the microglial transcriptome at the single-cell level in various mouse models of amyloid deposition have identified the induction of microglial subtypes including disease-associated microglia and activated response microglia [67, 68]. These microglial subtypes share similar transcriptome profiles—for example, increased expression of AD risk genes including *APOE* and *TREM2* as well as genes associated with lysosomal pathways—and are co-localized with Aβ plaques. These findings suggest that as AD progresses, microglia gradually adopt an activated state in response to the deterioration of the central nervous system microenvironment.

Interestingly, the activation status of microglia in AD is highly dynamic and reversible. For example, upon cytokine activation in AD, alternative activation states of microglia can be induced, such as anti-inflammatory phenotypes by NLRP3 inhibition or IL-33–responsive microglia by IL-33 activation [26, 27]. However, it remains unclear how these activated microglia differentiate from their precursor cells. Given that microglia robustly react to stimuli (i.e., within 1-h post-activation) [69, 70], comprehensive time-course profiling of the microglial transcriptome and functional characterization are essential to understand the molecular mechanisms underlying cytokine-induced microglial activation and its beneficial effects in AD.

**Fig. 2:**
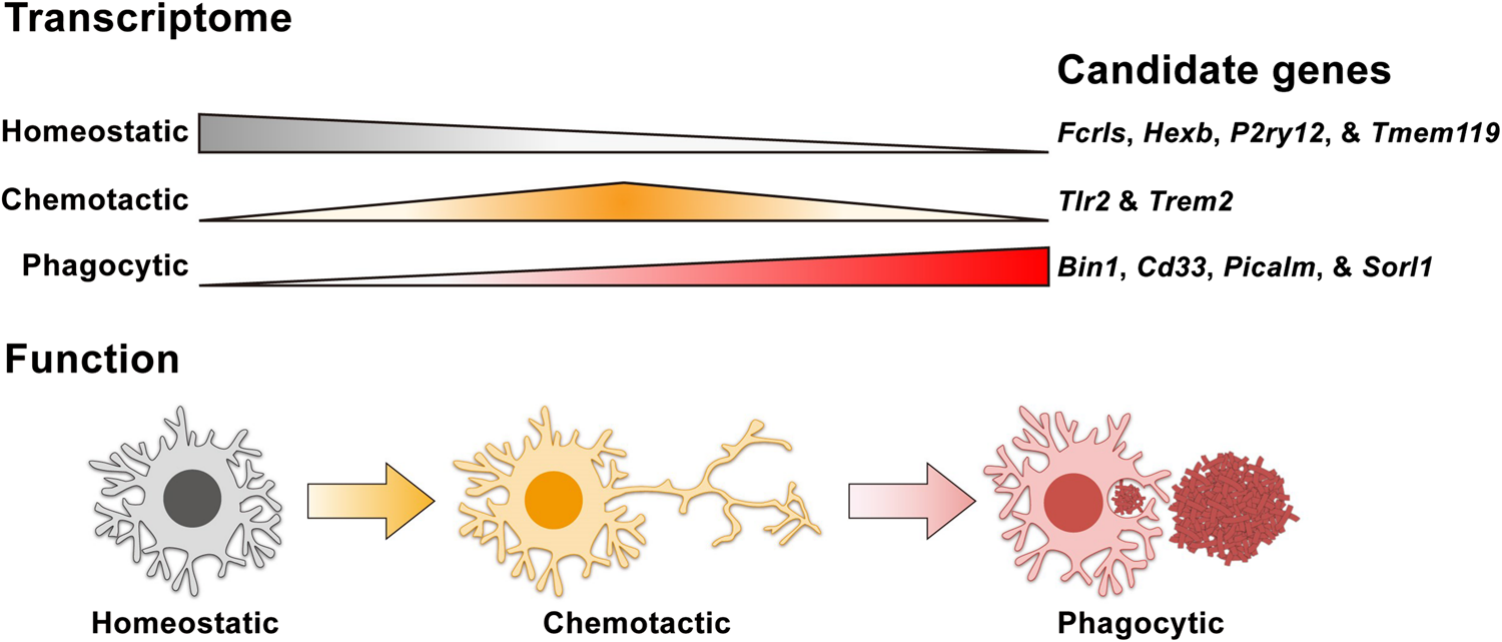
Proposed model of the stepwise microglial state transition stimulated by cytokine signaling. Colored bars indicate the expression level of each microglial transcriptomic signature during cytokine-induced state transition, with thicker bars indicating higher expression

Despite these unknowns, by summarizing the microglial phenotypes observed from the abovementioned studies, we propose a stepwise state transition model to delineate the beneficial cytokine-induced response of microglia in AD. Upon cytokine activation, microglia transcriptomically and functionally transition from a homeostatic state to a chemotactic state and finally to a phagocytic state in AD (Fig. 2). Here, we further discuss the two key states of the stepwise state transition of microglia—the chemotactic and phagocytic states—as well as their molecular regulators.

### Chemotactic state

Chemotaxis is the directional migration of a cell towards a particular ligand. To achieve this, microglia express specific surface receptors that sense their corresponding ligands. Given that transcriptomic profile reprogramming drives the functional state of microglia, the enhanced Aβ chemotaxis upon cytokine activation is likely due to increased expression of surface receptors that mediate the sensing of Aβ and its associated proteins such as ApoE [71, 72]. Several cell-surface receptors including TLR2 and TREM2 regulate the interactions of microglia with Aβ and ApoE [73–76]. Genetic ablation, particularly that of TREM2, impairs the recruitment of microglia towards Aβ as well as ApoE-dependent transcriptomic reprogramming of microglia (Fig. 2). Moreover, unpublished data from our laboratory indicate that IL-33 induces a repertoire of Aβ-sensing receptors shortly after administration in APP/PS1 mice. These findings collectively suggest that cytokine signaling can induce the expression of Aβ-sensing receptors and regulate microglial chemotaxis towards Aβ.

### Phagocytic state

Once microglia reach Aβ plaques, they mediate Aβ clearance via phagocytosis (Fig. 2). Similar to chemotaxis, the phagocytic capacity of microglia is also regulated by their transcriptomic signature, which determines the expression of phagocytic receptors, trafficking molecules, and protein-degrading enzymes. For example, *CD33*, an AD risk gene that encodes a sialic acid-binding immunoglobulin-like lectin, can inhibit the microglial uptake of neurotoxic Aβ_42_ [77]. Once Aβ is internalized, phagocytic vesicles containing Aβ enter lysosomal pathways, which leads to Aβ degradation. Interestingly, many AD risk genes such as *BIN1*, *PICALM*, and *SORL1* are important regulators for the trafficking of phagocytic vesicles [78]. While these findings show that a gene signature related to phagolysosomal pathways is crucial for the regulation of microglial phagocytosis, it remains largely unclear if cytokines control the phagocytic capacity of microglia by modulating the expression of such pathways in AD.

Therefore, additional detailed investigations are required to delineate how the gene signature of each microglial state contributes to the corresponding functions of microglia in AD. As such, we propose a stepwise state transition model to demonstrate the cytokine-stimulated microglial response in AD in which microglia transition from a homeostatic to a chemotactic state and finally to a phagocytic state.

## Concluding remarks

Impaired Aβ clearance is strongly associated with the pathogenesis of late-onset AD, which accounts for over 95% of AD cases. Therefore, enhancing microglial Aβ clearance is a promising therapeutic approach for AD. While targeting the cytokine signaling pathways discussed herein has great therapeutic potential, these pathways also trigger strong immune responses in the peripheral system, which limits their translational potential. Therefore, it is essential to understand the molecular and cellular bases of such beneficial outcomes—especially how these pathways promote the state transition and clearance activity of microglia. Indeed, as illustrated above, the microglial state transition is controlled at multiple levels by extracellular signals, signal transducers, transcription factors, and transcriptomes, suggesting that there are multiple targets for manipulating microglial states at each level. Accordingly, in-depth investigations of how the interplay among signaling transducers and transcription factors controls the microglial state transition in AD are required to identify precise molecular targets for enhancing the microglial state transition and restoring brain homeostasis in AD.
